# Changes in the secretome of tri-dimensional spheroid-cultured human mesenchymal stem cells in vitro by interleukin-1 priming

**DOI:** 10.1186/s13287-017-0753-5

**Published:** 2018-01-17

**Authors:** Elena Redondo-Castro, Catriona J. Cunningham, Jonjo Miller, Helena Brown, Stuart M. Allan, Emmanuel Pinteaux

**Affiliations:** Division of Neuroscience and Experimental Psychology, School of Biological Sciences, Faculty of Biology, Medicine and Health, University of Manchester, Manchester, UK

**Keywords:** Spheroid, 3D culture, Mesenchymal stem cell, Inflammation, BV2 cells, Microglia

## Abstract

**Background:**

Mesenchymal stem cells (MSCs) are one of the most promising candidates for the treatment of major neurological disorders. Desirable therapeutic properties of MSCs include reparative and regenerative potential but, despite their proven safety, the efficacy of MSCs remains controversial. Therefore, it is essential to optimise culture protocols to enhance the therapeutic potential of the MSC secretome. Here we aimed to: assess the increase in secretion of cytokines that may induce repair, regeneration, or immunomodulation when cultured in three dimensions; study the effect of interleukin (IL)-1 priming on two- (2D) and three-dimensional (3D) cultures of MSC; and evaluate the potential use of the modified secretome using microglial-MSC co-cultures.

**Methods:**

We established a 3D spheroid culture of human MSCs, and compared the secretome in 2D and 3D cultures under primed (IL-1) and unprimed conditions. BV2 microglial cells were stimulated with lipopolysaccharide (LPS) and treated with spheroid conditioned media (CM) or were co-cultured with whole spheroids. Concentrations of secreted cytokines were determined by enzyme-linked immunosorbent assay (ELISA). Protein arrays were used to further evaluate the effect of IL-1 priming in 2D and 3D cultures.

**Results:**

3D culture of MSCs significantly increased secretion of the IL-1 receptor antagonist (IL-1Ra), vascular endothelial growth factor (VEGF), and granulocyte-colony stimulating factor (G-CSF) compared with 2D culture, despite priming treatments with IL-1 being more effective in 2D than in 3D. The addition of CM of 3D-MSCs reduced LPS-induced tumour necrosis factor (TNF)-α secretion from BV2 cells, while the 3D spheroid co-cultured with the BV2 cells induced an increase in IL-6, but had no effect on TNF-α release. Protein arrays indicated that priming treatments trigger a more potent immune profile which is necessary to orchestrate an effective tissue repair. This effect was lost in 3D, partly because of the overexpression of IL-6.

**Conclusions:**

Increased secretion of anti-inflammatory markers occurs when MSCs are cultured in 3D, but this specific secretome did not translate into anti-inflammatory effects on LPS-treated BV2 cells in co-culture. These data highlight the importance of optimising priming treatments and culture conditions to maximise the therapeutic potential of MSC spheroids.

## Background

Mesenchymal (stromal) stem cells (MSCs) are an attractive candidate for use in therapies for the treatment of major central nervous system (CNS) conditions such as stroke, spinal cord injury, or amyotrophic lateral sclerosis [1–5]. Key features of MSCs include their ability to sense and migrate toward tumours [6] and inflamed, ischaemic, or injured tissues [7]. Additionally, MSCs can cross the blood-brain barrier [8] and secrete a wide variety of cytokines and trophic mediators [2, 9, 10] that induce tissue repair by activating endogenous protective mechanisms [11, 12]. These effects are achieved with a low engraftment rate and low rejection response from the host tissue, which confers MSCs a uniquely high level of safety [1–3]. Nevertheless, since many aspects of MSC biology are still unknown, efforts are being made to optimise culture conditions to fully exploit their therapeutic potential.

Our previous study demonstrated that preconditioning treatments of MSCs in two-dimensional (2D) culture with interleukin (IL)-1 enhances the secretion of pro-trophic molecules, such as granulocyte-colony stimulating factor (G-CSF) [13]. Furthermore, conditioned media (CM) from IL-1-primed MSCs modified the inflammatory response of microglial cells after lipopolysaccharide (LPS) stimulation, as demonstrated by a reduced secretion of inflammatory and pro-apoptotic molecules (such as IL-6 and G-CSF) and tumour necrosis factor (TNF)-α. This confirmed the use of 2D-MSCs as modulators of inflammation, as previously reported [10, 14]. Following on from our previous study, and with the aim of inducing a greater change in the MSC secretome, we assessed the effect of culturing cells in three dimensions and the effect of priming treatments in these MSC spheroids. MSCs express different secretomes when cultured in three dimensions, such as increased wound healing features and angiogenesis [15–19] and enhanced anti-inflammatory potential when grown under three-dimensional (3D) conditions [18, 20]. Three-dimensional cultures provide a more physiological environment for cells, and trigger enhanced expression of other desirable phenotypes, such as improved MSC stemness and increased survival post-transplantation [18]. Spheroid formation can be considered as an optimisation of MSC culture to enhance their therapeutic potential [17, 18, 21], and MSC spheroid treatments have already been tested with moderate success in bone regeneration [22, 23] and in inflammatory and ischaemic models, such as colitis and stroke [19, 24].

Here we show for the first time that, when cultured under 3D conditions, MSCs show marked increased secretion of the IL-1 receptor antagonist (IL-1Ra), vascular endothelial growth factor (VEGF), and G-CSF, the latter being further increased by priming with IL-1. This enhanced anti-inflammatory phenotype did not, however, translate into an anti-inflammatory effect of MSCs on LPS-mediated cytokine release in BV2 cells in co-culture. Cytokine arrays revealed that priming of 2D cells induced an overexpression of proteins related to inflammatory and immune responses, but not in 3D cultures. Primed 3D-MSCs not only modified their secretome and secreted some anti-inflammatory cytokines, but also induced an increase in IL-6, and a decrease in IL-10 and CCL22. On the other hand, priming of spheroids induced an increase in the secretion of matrix metalloproteinases (MMPs), key molecules for further tissue repair and angiogenesis. These findings highlight the importance of optimising culture methods and priming protocols to maximise the therapeutic potential of MSCs.

## Methods

### Cell cultures

#### Mesenchymal stem cells (MSCs)

Human bone marrow-derived MSCs were purchased from 3H Biomedical (Sweden). Cells (harvested from a 22-week fetus) were differentiated into osteocytes or adipocytes using commercial kits (Millipore, UK) and characterised by the presence or absence of specific surface markers (CD73, CD90, CD105, CD34, CD11b, CD19, CD45, and HLA-DR), the minimal criteria for identifying MSCs [25].

Culture flasks (Corning, UK) were coated with 0.1% gelatin in phosphate-buffered saline (PBS) overnight at 37 °C, and washed with PBS prior to cell seeding. Cells were expanded and cultured in MesenPRO RS® medium (Invitrogen, UK) supplemented with 1% penicillin/streptomycin and 2 mM glutamine (Sigma-Aldrich, UK). Medium was changed every 4–5 days until cultures reached 70–80% confluency. Cells were then detached using 0.5% trypsin-EDTA (Sigma-Aldrich, UK), counted, and split into different culture flasks, and further cultured as described above. Cells used for experiments were obtained from passage 5–6 and seeded at 13,000 cells/cm^2^ (for the 2D cultures),

#### 3D spheroid formation

**Fig. 1:**
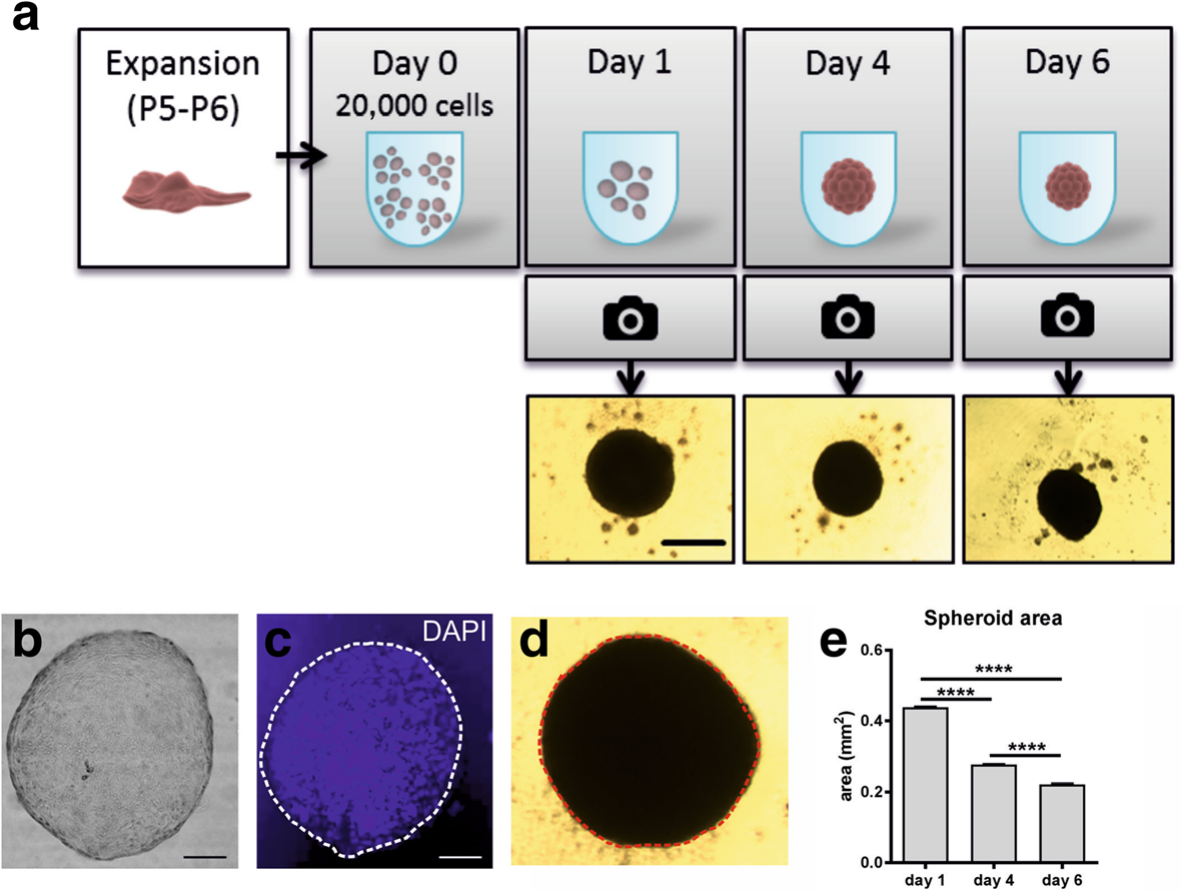
Formation and measurements of spheroids. **a** Scheme of the formation of spheroids, with representative images at different times of growth. **b** Haematoxylin and eosin staining of a 20-μm section of a spheroid, in a bright field image. **c** Immunostaining of the same section using DAPI, indicating a compact cellular structure. **d** Spheroids were measured at different time points by measuring the maximum sectional area, indicated in the image as a dashed line. **e** Areas of main spheroids (mean + SEM) were larger at early time points and decreased with time, indicating the compaction of the spheroid. Scale bars = 500 μm in **a**, 200 μm in **b–d**. ****p < 0.001. P passage

MSCs were harvested at passage 5–6 using 0.5% trypsin-EDTA, and sub-cultured in low-binding plates (Nunc International, Japan) using 50 μl MesenPRO RS®. The number of cells used in each spheroid (20,000 cells) was slightly higher than that used in 2D cultures, since not all cells formed spheroids. Spheroids were considered fully formed after 5 days in vitro [26]. Images of spheroids were taken with an inverted microscope (Olympus CK X31), using a Moticam 2300 camera coupled to Motic Images Plus 2.0 ML software at different time points to monitor spheroid formation. Spheroid size was estimated from the calculation of area and perimeter of the picture showing maximum sectional area (Fig. 1d). All clusters of cells with an area inferior to 0.15 mm^2^ were not considered spheroids and were therefore excluded in the measurements. The area was measured using ImageJ software (U.S. National Institutes of Health, Bethesda, Maryland, USA), and 25–30 spheroids for each treatment were included in the measurement (total *n* = 131 spheroids).

To confirm their physical structure, spheroids were fixed in 4% paraformaldehyde, cryopreserved with 30% glucose in phosphate buffer, and stained with haematoxylin and eosin (Fig. 1b) before being sectioned at 20 μm for DAPI staining (Fig. 1c).

#### Microglial cells (BV2s)

BV2 cells, an immortalised murine microglial cell line, were purchased from ATCC (UK) and cultured in RPMI-1640 medium (Sigma-Aldrich, UK) supplemented with 10% fetal bovine serum (FBS; Invitrogen, UK) and 1% penicillin/streptomycin (Sigma-Aldrich, UK) until 70–80% confluent. Cells were detached with 0.5% trypsin-EDTA (Sigma-Aldrich, UK), counted, and seeded at a density of 13,000 cells/cm^2^.

### Cell treatments

#### Priming experiments in 2D versus 3D

Cells were treated (24 h after plating for 2D cultures or 5 days in vitro after plating for 3D cultures) with human recombinant IL-1α, IL-1β, TNF-α, or interferon (IFN)-γ at a final concentration of 10 ng/ml (all from R&D Systems, UK). After 24 h, supernatants were collected and assayed for key human cytokines. As spheroid formation requires a small amount of media, cytokine concentrations from 2D and 3D cultures were normalised by the volume of supernatant collected from the wells at the end of the experiment (~50 μl in 3D, ~500 μl in 2D). The initial number of cells was also used as a normalisation factor. Three independent cultures were included, with at least three technical replicates per condition.

#### Co-culture experiments: BV2 and 3D conditioned media (CM)

MSC spheroids cultured for 5 days in vitro were treated for 2 h with 10 ng/ml IL-1α or IL-1β, and then washed twice with PBS and placed back in the low-binding plate with fresh media. Priming time was reduced to 2 h as shorter times were sufficient to induce changes in cytokine secretion (data not shown). Supernatants were collected 24 h later for further analysis of secreted cytokines by enzyme-linked immunosorbent assay (ELISA).

In parallel, 25,000 BV2 cells were seeded, cultured for 24 h, and then treated for 2 h with bacterial LPS (1 μg/ml, serotype *E. coli* 0127:B8, Sigma-Aldrich, UK). After LPS treatment, cells were washed twice with PBS and the spheroid-CM was added to BV2 cells, diluted 1/10 in MesenPRO RS®. Supernatants were collected 24 h later and further analysed for the presence of mouse and human cytokines by ELISA. Final volumes in the wells were matched in each condition; therefore, no normalisation was carried out in this experiment. Three independent cultures were included, with at least three technical replicates per condition.

#### Co-culture experiments: BV2 and whole 3D spheroids

MSCs and BV2 cells were seeded and treated as described; however, after the priming treatment with IL-1, MSC spheroids were washed twice with PBS and placed into a transwell insert (0.4-μm pore size; Greiner Bio One, UK) that had been previously equilibrated in RPMI media overnight. Inserts were placed on top of BV2 cells for 24 h, after which supernatants were collected and further analysed for the presence of mouse and human cytokines by ELISA. Another set of spheroids was primed (or left unprimed) as previously described and maintained in inserts in the absence of BV2 cells. Final volumes in the wells were matched in each condition; therefore, no normalisation was carried out in this experiment. Three independent cultures were included, with at least three technical replicates per condition.

### ELISA

The levels of VEGF, G-CSF, IL-6, TNF-α, and IL-10 were quantified by ELISA using mouse (m) or human (h) DuoSet® kits (R&D Systems, UK) according to the manufacturer’s instructions. IL-1Ra levels were measured using an ELISA kit from Peprotech (UK) combined with external standards prepared using recombinant human IL-1Ra (National Institute for Biological Standards and Controls, NIBSC, UK). For each assay, protein levels were calculated against a four-parameter logistic (4-PL) curve fit. Quantification limits were ~30 pg/ml for all the cytokines measured (except TNF-α, ~60 pg/ml), and no statistical analyses were carried out for values below this limit.

### Membrane arrays

A human cytokine antibody array (Abcam, UK) was used to compare the secretion of cytokines secreted by MSCs in 2D and 3D, under primed and unprimed conditions. Supernatants from 2D and 3D were concentrated and diluted (respectively) up to four times, so that results from the arrays were directly comparable.

A human MMP array was used to assess the differences in secretion of tissue remodelling proteins in 3D cultures. Supernatants from at least two independent experiments were pooled to reach 1 ml of supernatant required for the membrane array.

For both arrays, densitometry data were obtained using ImageJ software, and normalised against the untreated values; therefore, the priming effect could be easily observable in 2D and 3D cultures. A PANTHER over-representation test [27] including all the analysed cytokines was performed against a human protein database to detect the main biological processes related to the overexpressed proteins.

### Statistical analysis

Cytokine concentrations are expressed as mean ± standard error of the mean (SEM). Data presentation and analysis was performed using GraphPad Prism software version 6.04 for Windows (California, USA). All differences were assessed by one-way analysis of variance (ANOVA). Data from 2D–3D comparisons were analysed after applying a linear logarithmic transformation. Holm Sidak post-hoc tests were performed if statistical significance was achieved (*p* < 0.05). Densitometry data were normalised versus the untreated conditions and differences were assessed by two-way ANOVA. Sidak’s multiple comparison tests were applied if statistical significance was achieved (*p* < 0.05).

## Results

### Characterization of MSCs

Cells were positively characterised as MSCs for their ability to differentiate into adipocytes and osteocytes and by the presence of CD90, CD105, and CD73 surface markers and the absence of CD34, CD11b, CD45, CD19, and HLA-DR, as stated by the International Society for Cellular Therapy [25] and as we have previously published [13].

### Assessment of spheroid formation

All wells containing cells generated at least one spheroid. At day 1, spheroids were visible in all wells (Fig. 1a) sometimes surrounded by individual cells. At day 4, most of the cells had been incorporated into spheroids, and at day 6 the number of individual cells surrounding the spheroids was markedly reduced. Spheroids were visualised as compact cellular structures (Fig. 1b, c), and were significantly smaller (measured as indicated in Fig. 1d) at day 6 compared to day 4 (Fig. 1e), indicating compaction as previously described [20, 28].

### MSCs display increased secretion of cytokines in 3D cultures

**Fig. 2:**
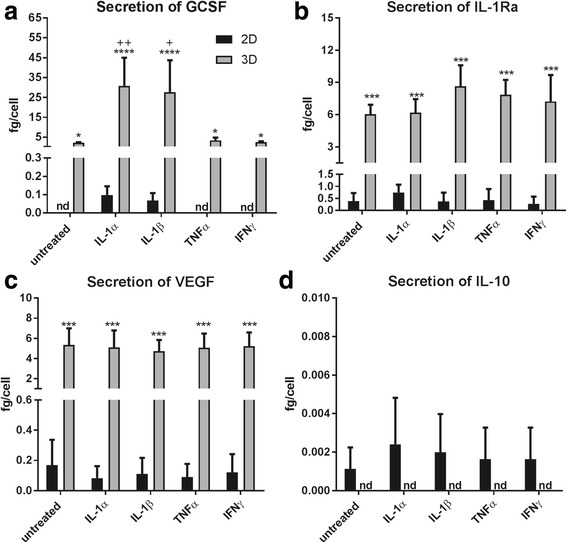
Secretion of different trophic factors and inflammatory mediators in two-dimensional (2D) and three-dimensional (3D) cultures. Cells cultured in 2D and 3D were primed with Interleukin (IL)-1α, IL-1β, tumour necrosis factor (TNF)-α, or interferon (IFN)-γ. ELISA measurements (normalized to the initial number of cells and the final volume in the wells after the treatments) indicate that cells in 3D secrete more granulocyte-colony stimulating factor (GCSF) (**a**), IL-1 receptor antagonist (IL-1Ra) (**b**), and vascular endothelial growth factor (VEGF) (**c**), but not IL-10 (**d**). Priming treatments were only effective in inducing the secretion of GCSF. *p < 0.05, ***p < 0.001, versus 2D; ^+^*p* < 0.05, ^++^*p* < 0.01, versus untreated. nd not detectable

The secretion of different cytokines was measured in supernatants of MSCs cultured in 2D and 3D. Cells in 2D secreted low concentrations of G-CSF, only detectable after priming treatment with IL-1 (Fig. 2a). Secretion of G-CSF was significantly increased by 20-fold in 3D cultures (up to ~2 fg/cell in untreated conditions; *p* < 0.05). Priming spheroids with IL-1α and IL-1β increased secretion of G-CSF sixfold (*p* < 0.0001, 2D versus 3D; *p* < 0.01, IL-1α versus untreated; *p* < 0.05, IL-1β versus untreated). TNF-α and IFN-γ treatment had no effect on G-CSF secretion in 2D or 3D cultured cells. Secretion of IL-1Ra from 2D cultured MSCs was unaffected by priming (Fig. 2b), but was significantly increased when cultured in 3D cultures (18-fold increase on average; *p* < 0.001, 2D versus 3D). Priming had no effect on IL-1Ra secretion in 3D cultures. VEGF secretion was strongly augmented in spheroids compared to 2D conditions (25-fold increase; *p* < 0.001; Fig. 2c). None of the priming treatments changed the secretion of VEGF, whether cells were in 2D or 3D culture. Secretion of IL-10 under 2D conditions was very low and not detectable when in 3D, and there was no effect of any priming treatment (Fig. 2d).

### Modulation of BV2 inflammatory responses

Given the enhanced anti-inflammatory phenotype, we hypothesised that the increased secretion of cytokines induced by the 3D culture of MSCs could exert anti-inflammatory effects on LPS-stimulated BV2s. Since only IL-1α and IL-1β priming induced enhanced secretion of G-CSF, only these cytokines were tested in further experiments.

#### Treatment of BV2 with conditioned media from MSC spheroids

**Fig. 3:**
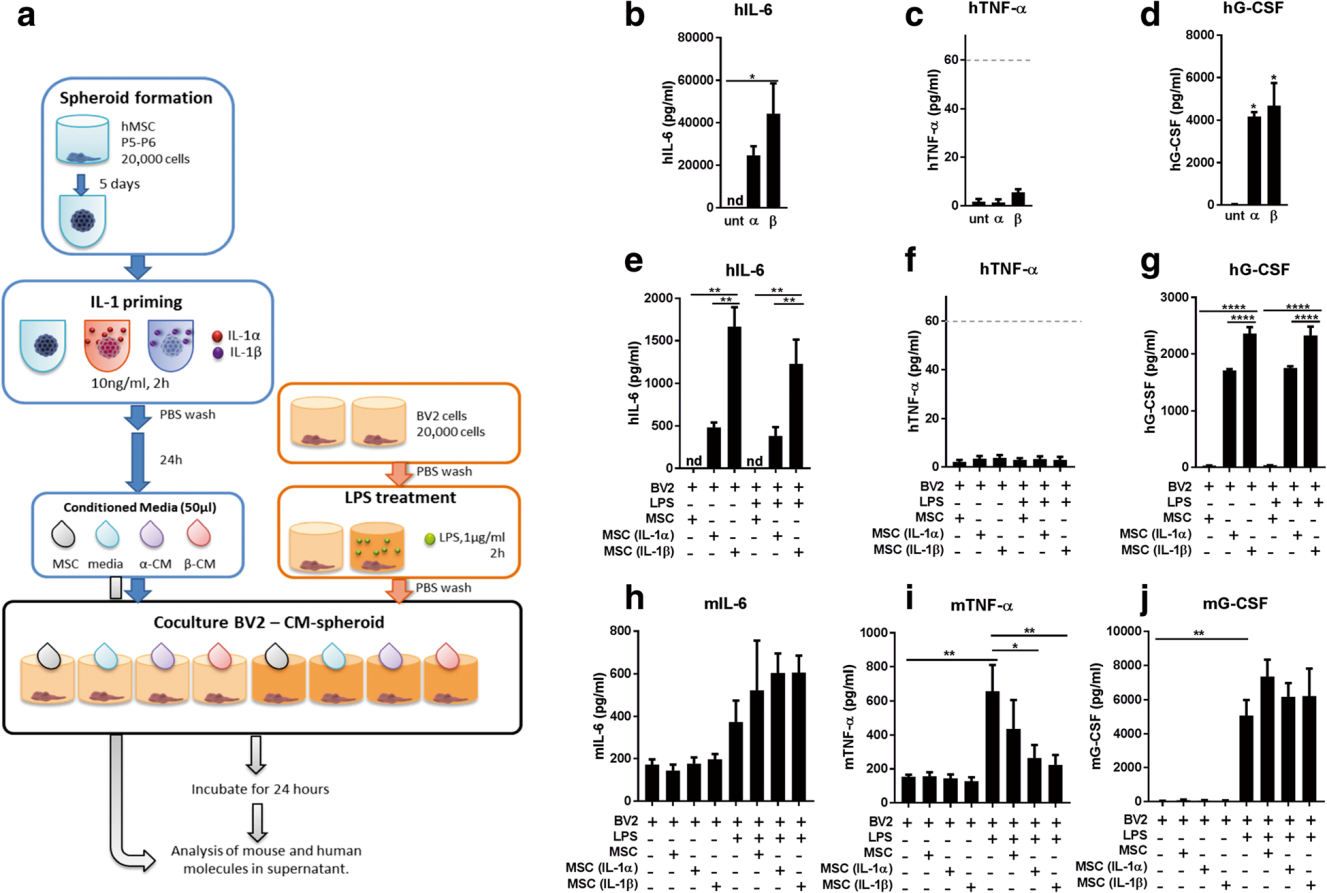
Treatment of BV2 using conditioned media (CM) from spheroids. **a** Mesenchymal stem cells (MSCs) were expanded and cultured in 3D to form spheroids. After interleukin (IL)-1 priming, conditioned media from spheroids were collected and added to BV2 cells previously treated with lipopolysaccharide (LPS). Human (h) and murine (m) cytokines were analysed from supernatants 24 h later. Measurements of human cytokines in the conditioned media (**b–d**) and in the supernatant from treated BV2s (**e–g**). Murine cytokines were also analysed from the supernatants (**h–j**). Results indicate that priming spheroids increased the secretion of IL-6 and granulocyte-colony stimulating factor (G-CSF). Analysis of the supernatant of the co-culture revealed a significant decrease in murine tumour necrosis factor (mTNF)-α after treatment with CM from primed spheroids. Dashed lines in **c** and **f** indicate the quantification limit for TNF-α ELISA. *p < 0.05, **p < 0.01, ***p < 0.001, ****p < 0.0001. nd not detectable, P passage, PBS phosphate-buffered saline, unt untreated

BV2 cells previously treated with LPS were treated with CM from primed spheroids (alongside appropriate controls, as shown in Fig. 3a). Priming of MSC spheroids induced a significant increase in hIL-6 secretion (Fig. 3b; *p* < 0.05) and hG-CSF (Fig. 3d; *p* < 0.05), but not hTNF-α (Fig. 3c), as measured in the 3D-CM.

When 3D-CM was added to BV2 cells, the concentrations of the same human cytokines were reduced after 24 h, indicating uptake or degradation by the BV2 cells. Human IL-6 was undetectable in untreated conditions, but was significantly increased after IL-1α and IL-1β priming (Fig. 3e; *p* < 0.01). The response of hG-CSF was similar to hIL-6 (Fig. 3g; *p* < 0.01, IL-1α/β versus untreated; *p* < 0.0001, IL-1α versus IL-1β). Levels of hTNF-α were below the quantification limit under all conditions (Fig. 3f, dashed line).

Finally, quantification of murine molecules in the supernatant showed a trend towards an increase in mIL-6 under LPS-treated conditions (Fig. 3h). Secretion of mTNF-α from BV2s (Fig. 3i) increased following LPS stimulation (greater than fourfold increase), and this was significantly reduced by treatment with CM from IL-1α- and IL-1β-primed spheroids (*p* < 0.05 and *p* < 0.01, respectively). Levels of mG-CSF (Fig. 3j) increased more than 100-fold with the LPS treatment (*p* < 0.01), and were not altered by CM treatment.

#### Co-culture of BV2 and MSC spheroids

**Fig. 4:**
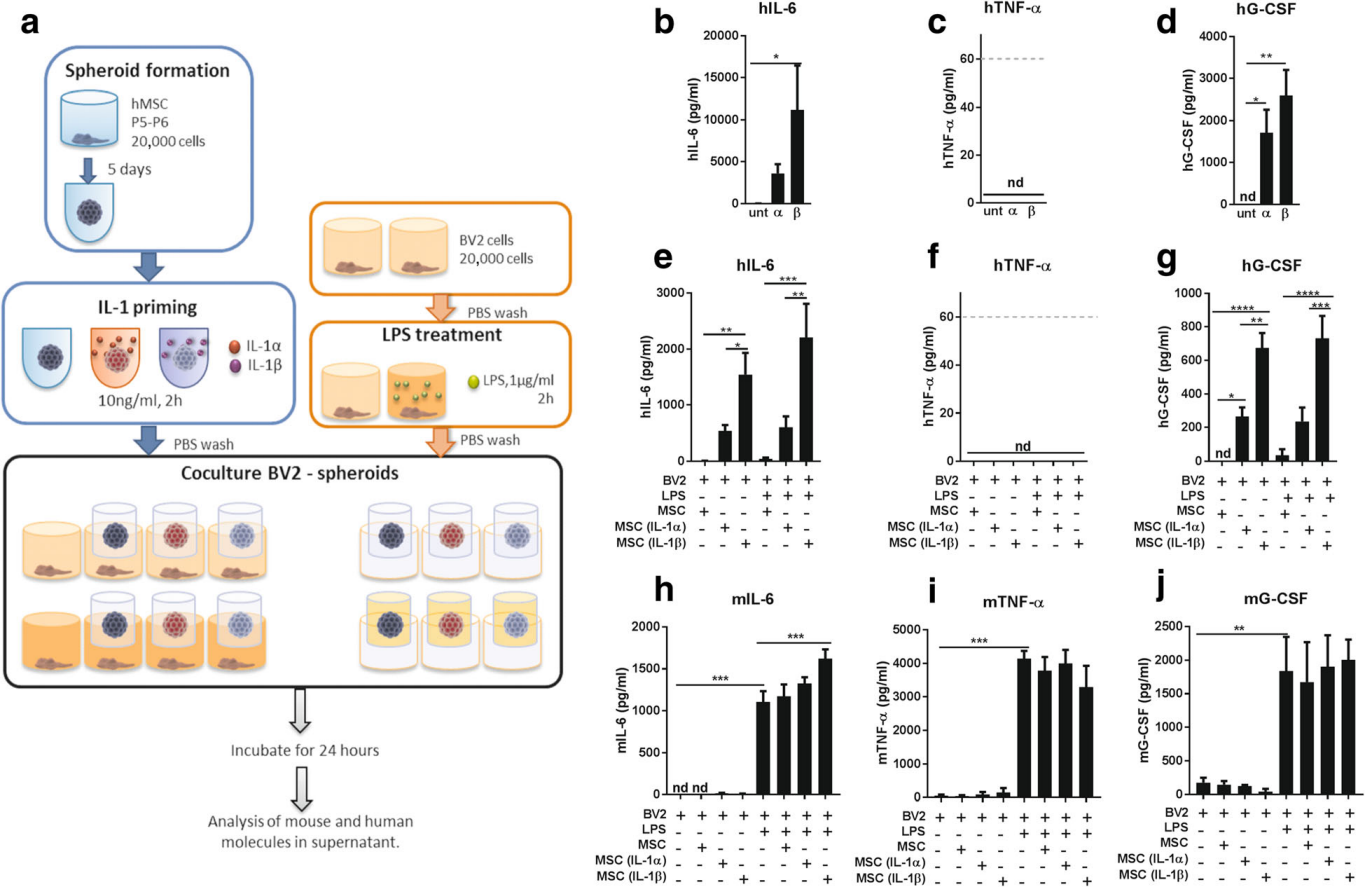
Treatment of BV2 using spheroids in inserts. **a** Mesenchymal stem cells (MSCs) were expanded and cultured in 3D to form spheroids, primed with interleukin (IL)-1 and co-cultured in inserts with BV2 cells previously treated with lipopolysaccharide (LPS). Measurements of human (h) cytokines on the conditioned media added to the BV2s (**b–d**), and in the conditioned media obtained from cells after 24 h in the inserts (**e–g**). Murine (m) cytokines were also analysed from the co-culture supernatants (**h–j**). Analysis of the supernatant of the co-culture revealed a significant increase in mIL-6 after adding conditioned media (CM) from IL-1β-primed spheroids. Dashed lines in **c** and **f** indicate the quantification limit for tumour necrosis factor (TNF)-α ELISA. *p < 0.05, **p < 0.01, ***p < 0.001, ****p < 0.0001. G-CSF granulocyte-colony stimulating factor, nd not detectable, P passage, PBS phosphate-buffered saline, unt untreated

To maximise the effect seen with the spheroid CM on the BV2 response to LPS, co-culture experiments with whole spheroids were conducted (including appropriate control experimental conditions, as depicted in Fig. 4a). Measurements of CM from spheroids placed in inserts for 24 h showed that priming treatments with IL-1 induced an increase in hIL-6, especially after IL-1β (Fig. 4b; *p* < 0.05), but not for hTNF-α which remained undetectable (Fig. 4c). Human G-CSF secretion was not detectable in untreated conditions, but significantly increased after IL-1α and IL-1β priming (Fig. 4d; *p* < 0.05 and *p* < 0.01, respectively). These results demonstrate a similar response to priming as in the previous experiment (Fig. 3b–d); however, lower concentrations of IL-6 and TNF-α were observed here, indicating that contact of the spheroid with the insert reduces the secretion of these cytokines from MSCs.

The concentration of these cytokines was consequently reduced when measured in the supernatant of the co-culture, indicating the uptake or break-up of human proteins by murine cells (Fig. 4e–g). Levels of IL-6 were significantly increased because of the priming (Fig. 4e; *p* < 0.05 and *p* < 0.01, versus untreated), as well as levels of G-CSF, especially after IL-1β treatment (Fig. 4g; at least *p* < 0.05, versus untreated,). TNF-α remained undetectable (Fig. 4f).

Treatment of BV2s with LPS induced a significant increase in the secretion of mIL-6 (*p* < 0.001), and co-culture with spheroids induced a higher secretion of IL-6 (statistically significant only in the case of IL-1β-primed spheroids; Fig. 4h; *p* < 0.001, versus LPS). Secretion of mTNF-α from BV2s was significantly increased following LPS stimulation (Fig. 4i; *p* < 0.001). Spheroid co-culture did not induce any change in TNF-α secretion. Similarly, mG-CSF significantly increased under all LPS-treated conditions (Fig. 4j; *p* < 0.01). The addition of primed spheroids had no effect on the secretion of mG-CSF.

### IL-1 priming induced the secretion of cytokines related to immune and inflammatory responses

**Fig. 5:**
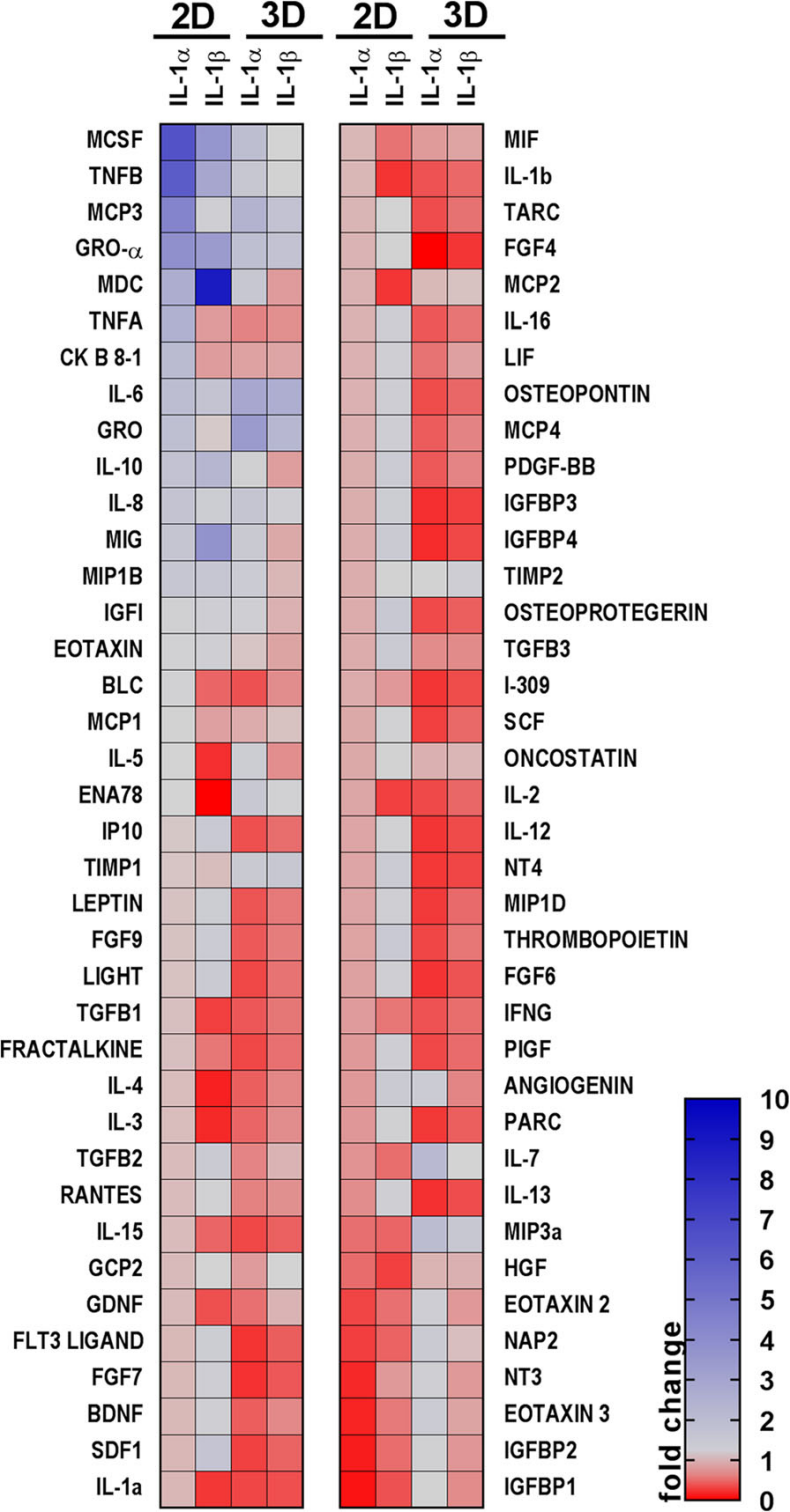
Heat map showing the cytokine concentrations secreted by MSCs cultured in two-dimensions (2D) and three dimensions (3D) after priming with IL-1α or IL-1β. Cytokines were analysed from the conditioned media of MSCs cultured in 2D and 3D, under untreated or primed conditions (IL-1α or IL-1β). Colours are assigned according to the relative scale of expression, ranging from 0 to 10, and representing fold-increase change versus the untreated condition

To evaluate possible key mediators involved in the effects seen in the BV2 culture, a protein array was performed including a wide variety of cytokines and trophic factors. Once data were normalised against the untreated condition, two-way ANOVA revealed that the effect of different culture conditions (2D or 3D) had a major effect (*p* < 0.05) on the change in protein secretion, rather than the priming treatments. Despite every treatment and condition inducing a unique combination of proteins being differentially secreted, only a few cytokines appeared upregulated under all the conditions (greater than twofold increase), such as macrophage-colony stimulating factor (M-CSF), TNF-β, CCL22, or growth-regulated protein alpha (GRO-α) (Fig. 5). The over-representation test [27] performed with these proteins indicated that these proteins were related to the orchestration of inflammatory and immune responses, affecting the function of other cell types such as macrophages or monocytes and inducing a higher response to cytokine signalling processes. More precisely, regulation of receptor activity, cytokine signalling, and inflammatory response were some of the terms that presented the smaller *p* values (*p* < 10^–10^; Fig. 5 and Tables 1 and 2), confirming the involvement of these over-expressed cytokines in the cited biological processes. Despite all these processes also seeming to be over-represented in the 3D secretomes, the increase in the expression of these cytokines was higher under the 2D conditions.

**Table 1:** Over-represented biological processes after interleukin (IL)-1 priming in two-dimensional and three-dimensional cultures

Biological process	*p* value
Regulation of receptor activity	5.93E-14
Cytokine-mediated signalling pathway	9.27E-14
Inflammatory response	2.64E-12
Cellular response to cytokine stimulus	5.19E-12
Response to cytokine	1.65E-11
Cell chemotaxis	4.83E-11

List of the biological processes that were over-represented in the analysis of the cytokine array, and their corresponding *p* value. The list of proteins analysed were compared against a human database using a PANTHER over-representation test

**Table 2:** Main cytokines upregulated after interleukin (IL)-1 priming in two-dimensional and three-dimensional mesenchymal stem cells

Cytokine	Name	Function
MCSF	Macrophage colony-stimulating factor	Induces haematopoietic stem cells to differentiate into macrophages. Promotes release of pro-inflammatory cytokines to induce immune and inflammatory responses
TNF-β	Tumour necrosis factor β or lymphotoxin-α	Regulation of cell survival. Immunostimulatory mediator
CCL7/MCP3	Chemokine (C-C motif) ligand 7 or monocyte-chemotactic protein 3	Monocyte attractant and regulator of macrophage functions
Gro-α CXCL1	Growth-regulated protein alpha	Cell proliferation, involved in inflammatory and immune responses
CCL22	C-C motif chemokine 22	Attractant of immune cells to inflammatory sites
TNF-α	Tumour necrosis factor α	Involved in inflammatory and immune responses
CK B 8-1 or CCL23	C-C motif chemokine 23	Chemotactic activity for monocytes, resting T lymphocytes, and neutrophils. Involved in responses to IL-1
IL-6	Interleukin-6	Inducer of acute phase response. Dual effects as pro- and anti-inflammatory molecules
IL-10	Interleukin-10	Anti-inflammatory. Inhibits the synthesis of interferon (IFN)-γ, IL-2, IL-3, TNF, and GM-CSF in macrophages
IL-8	Interleukin-8	Chemotactic for neutrophils and T cells
MIG/CXCL9	Monokine induced by gamma interferon/C-X-C motif chemokine 9	Affects activation state of cells involved in immune and inflammatory responses
G-CSF	Granulocyte colony-stimulating factor	Anti-apoptotic, involved in immune and inflammatory responses

Names of the main cytokines modified by priming treatments, with some of their main functions described. These cytokines were the most upregulated after priming with IL-1. All function terms have been obtained from the UniProt database

### IL-1β regulates expression of MMPs in 3D-MSCs

**Fig. 6:**
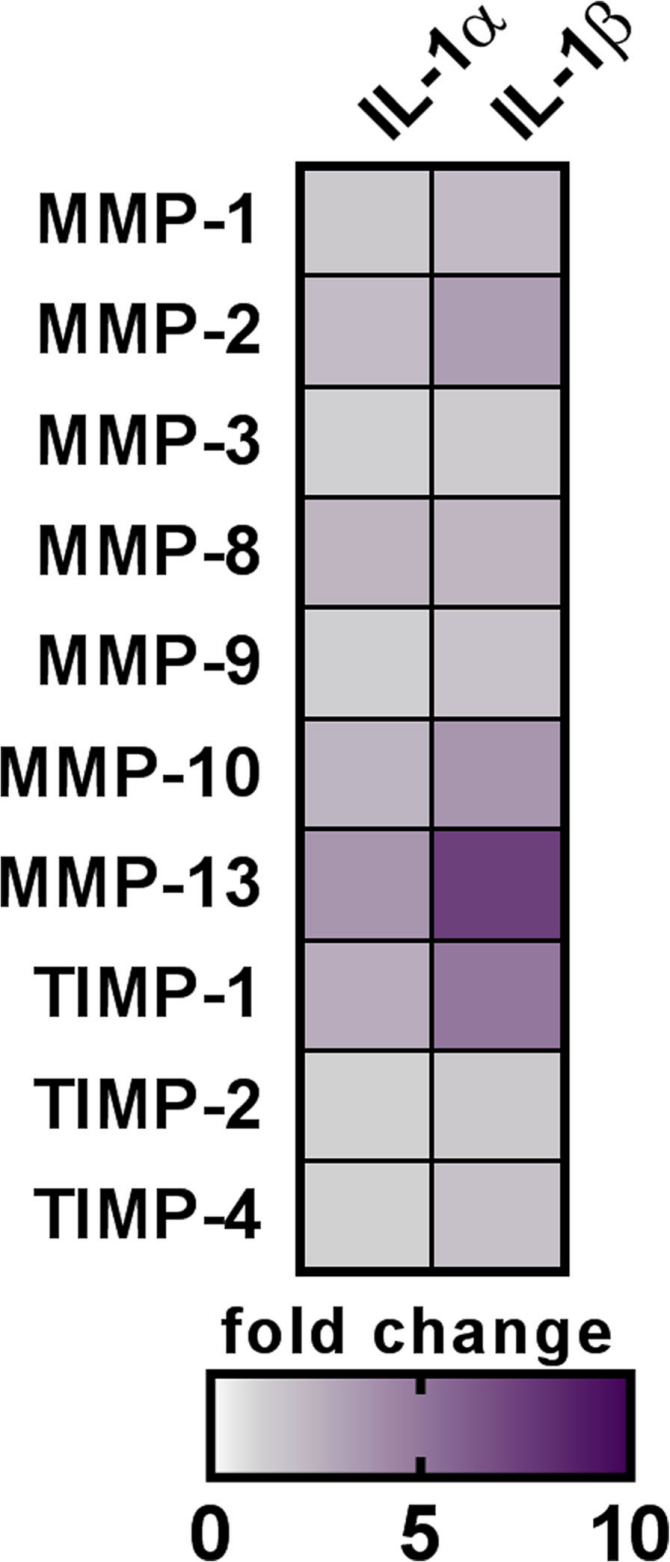
Expression of tissue remodelling molecules in 3D-MSC-conditioned media. Several matrix metalloproteinases (MMPs) and tissue inhibitors of metalloproteinases (TIMPs) were analysed from the conditioned media of 3D-MSC cultures (untreated or primed with interleukin (IL)-1α or IL-1β). Expression changes are expressed as fold-increase versus the untreated values and coloured according to the relative scale of expression ranging from 0 to 10

Conditioned media from 3D-MSCs (primed or unprimed) were used to analyse the expression of tissue remodelling proteins. MMP protein arrays showed increases in MMP-2, MMP-10, MMP-13, and tissue inhibitor of metalloproteinases (TIMP-1) in IL-1α- and IL-1β-primed MSC spheroids (Fig. 6). MMP-13 and TIMP-1 showed the greatest increases, especially after IL-1β priming.

## Discussion

Modulation of the secretome of MSCs is an essential step towards achievement of the full therapeutic potential of MSCs. Despite the enhancement in the secretion of cytokines and trophic factors that has been previously described in 3D-MSCs [18, 29, 30], there are only a few studies in vitro that tested this potential in macrophages [20] and cancer cells [21]. The efficacy of 3D-MSCs in vivo has not been fully proven, with just a few studies reporting their pro-angiogenic effects [15, 16], and only a couple of secretome modulation approaches have already been proven to be successful in in-vivo models of cerebral ischaemia [17, 19].

Here, we show that 3D culture modifies the secretome of human MSCs, leading to an enhanced secretion of G-CSF and IL-1Ra, key molecules involved in tissue repair and modulation of inflammation. As we have recently demonstrated that CM from 2D-MSCs exerts potent anti-inflammatory effects on LPS-treated BV2 microglial cell cultures [13], in this present study we hypothesised that an enhanced anti-inflammatory phenotype of 3D-MSCs could exert more potent anti-inflammatory effects on LPS-treated BV2 cells. Specifically, we tested whether the secretion of inflammation markers in BV2 cells could be modified by treating them with 3D-CM or co-culturing with whole MSC spheroids placed in inserts.

Only IL-1-primed spheroid CM resulted in decreased TNF-α secretion from LPS-treated BV2s, indicating the importance of priming treatments for modulating responses to inflammation in other cell types. This effect was lost when the whole spheroids were co-cultured with BV2s, as TNF-α secretion was not reduced and an increase in IL-6 was observed instead, particularly in the presence of IL-1β-primed spheroids.

To explain these results, more cytokines were included in a secretome analysis, and it was revealed that IL-1 priming also increased the secretion of several proteins involved in the inflammatory and immune response and the recruitment of immune cells (including GRO-α, MCSF, CCL22, and CCL7), and that this increase was more marked in 2D cells than in spheroids. This effect is achieved by secreting a wide variety of cytokines [9] that would act in other cells, mostly immune cells [31, 32], but not necessarily microglia (or BV2 cells in this case). In other words, IL-1 priming might be enhancing the ability of MSCs to induce a microenvironment that permits the repair of injured and inflamed tissues. Despite the upregulation of some proteins under both 2D and 3D conditions, the magnitude of the change was smaller in 3D conditions. This attenuated effect of IL-1 priming in 3D-MSCs may be due to the fact that the IL-1 signalling cascade is already upregulated due to the spheroid formation process itself [28], or to the fact that only the cells on the outer surface of the spheroids are exposed to these treatments. Besides, 3D-MSCs not only modified their secretome towards the release of more anti-inflammatory mediators but also induced other changes that may be detrimental in our co-culture model, such as the increase in IL-6 and the decrease in IL-10 and CCL22. We cannot discard here the dual role of certain cytokines such as IL-6 or G-CSF [33–35].

We also assessed the secretion of matrix metalloproteinases (MMPs) involved in tissue remodelling and repair, and which are believed to also have a dual role in neurological conditions such as stroke. While they mediate tissue injury in the acute phase of ischaemic stroke, their actions may well be beneficial during the recovery phase [36, 37]. In fact, MMP-9 and MMP-13 are involved in lesion growth [37], and MMP9 is also associated with increases in lesion volume and neurological deficits [38]. Priming of 3D-MSC spheroids leads to increased levels of MMP-13, particularly after treatment with IL-1β, and so may not have a therapeutic benefit if given in the acute phase after stroke. On the other hand, MMPs may have a valuable role in the recovery phase by contributing to neurovascular remodelling [36, 39] and by increasing of the bioavailability of VEGF [40]. Furthermore, TIMP-1 has anti-apoptotic effects and may be neuroprotective when given after stroke [41, 42].

Despite being promising in preclinical and in-vitro preparations, MSC spheroids are no more effective than their 2D counterparts [43, 44], confirmed by the fact that there are few published reports showing positive effects of MSC spheroids.

Nevertheless, MSC spheroids still represent a potential as a therapeutic option since they become less entrapped in the lungs and the capillaries when transplanted, and thus can survive for longer [28, 45], and they offer the chance of obtaining a unique combination of anti-inflammatory and immunomodulatory factors [31]. However, despite presenting all these desirable characteristics, the efficacy of spheroid MSCs still remains unproven. Indeed, treatment with CM (instead of the whole spheroid) may be a more effective treatment, maintaining anti-inflammatory and immune modulatory effects but mitigating the risks associated with a cell transplantation. Future studies should focus on assessing the efficacy of these treatments on in-vivo models of inflammation.

However, it is crucial to continue to increase our knowledge of the basic biology of MSCs, as priming treatments and changes in culture conditions can have great effects on the MSC secretome [13, 31, 45] and may increase their efficacy in the treatment of CNS conditions.

## Conclusions

Here, we describe how MSCs secrete more anti-inflammatory, pro-trophic, and pro-angiogenic factors when they are cultured under 3D conditions. However, this is not translated into a reduction in inflammatory responses in in-vitro cultures of BV2 cells, and the secretome analysis indicates that MSCs have a potential role as great orchestrators of inflammatory and immune responses by acting on other cell types, and that this phenotype can be modulated by IL-1 priming, especially in MSCs cultured under 2D conditions. These results highlight the importance of improving our understanding of MSC biology under different culture and priming conditions to optimise their potential therapeutic use.

## Abbreviations

2D: Two-dimensional
3D: Three-dimensional
ANOVA: Analysis of variance
CM: Conditioned media
CNS: Central nervous system
ELISA: Enzyme-linked immunosorbent assay
G-CSF: Granulocyte-colony stimulating factor
GRO-α (CXCL1): Growth-regulated protein alpha
IFN: Interferon
IL: Interleukin
IL-1Ra: Interleukin-1 receptor antagonist
LPS: Lipopolysaccharide
M-CSF: Macrophage-colony stimulating factor
MMP: Matrix metalloproteinase
MSC: Mesenchymal stem cell
PBS: Phosphate-buffered solution
SEM: Standard error of the mean
TIMP-1: Tissue inhibitor of metalloproteinases
TNF: Tumour necrosis factor
VEGF: Vascular endothelial growth factor
